# Embedding structural and social determinants of cardiovascular health into medical education: a systematic review of pedagogical frameworks

**DOI:** 10.3389/fmed.2026.1860983

**Published:** 2026-07-13

**Authors:** Omar Dabash, Michael J. Daly

**Affiliations:** 1 School of Medicine, RCSI University of Medicine and Health Sciences, Dublin, Ireland; 2 Department of Cardiology, Connolly Hospital, Dublin, Ireland

**Keywords:** cardiovascular disease, health equity, health professions education, pedagogical approaches, social determinants of health, structural competency

## Abstract

**Background:**

Cardiovascular disease remains the leading cause of preventable death globally, yet its burden is unequally distributed across social and structural lines. Given its unique clinical and social richness, cardiovascular disease represents a compelling vehicle through which health professions learners can develop the competencies needed to address these disparities in practice. This review aims to synthesise existing evidence on how cardiovascular disease is used as a teaching vehicle for structural and social competency in health professions education, and to evaluate the effectiveness of such interventions among health professions learners.

**Methods:**

Six databases were searched for studies describing educational interventions incorporating cardiovascular disease to teach structural and social determinants of health to health professions learners. Data on learner type, CVD topic, SDOH content, pedagogical approach, learner outcomes, and methodological quality were extracted.

**Results:**

Five studies met the inclusion criteria, all conducted in the United States, spanning medical students, pharmacy students, health science students, and cardiovascular fellowship trainees. Hypertension, myocardiaxl infarction, and women’s cardiovascular disease served as the primary teaching topics, with race, racism, and socioeconomic status as the most consistently addressed structural determinants. Interventions ranged from single sessions to a multimodal longitudinal fellowship curriculum. Three studies demonstrated statistically significant pre-to-post improvements in learner knowledge, attitudes, and structural understanding; one produced high-quality written reflections in 90%–96% of students; and one reported positive qualitative feedback without formal quantitative assessment.

**Conclusion:**

The available evidence suggests that cardiovascular disease is a feasible and promising teaching vehicle for structural and social competency, but current evidence is limited by small study numbers, US-only settings, heterogeneous interventions, and limited long-term outcome data. The results support investment in CVD-focused interventions, especially if longitudinal and experiential, to develop a more structurally competent health workforce.

**Systematic review registration:**

PROSPERO: CRD420251231266.

## Introduction

Cardiovascular disease (CVD) remains the leading cause of morbidity and mortality globally, yet despite advances in prevention, diagnostics, and treatment, the burden of CVD is unequally distributed across social and structural lines (1, 2). The traditional biomedical model taught in medical education has historically concentrated on individual clinical risk factors while neglecting the upstream structural and social forces that drive cardiovascular health outcomes (3, 4). In the United States, Black adults experience hypertension at among the highest rates in the world, with earlier onset, greater severity, and worse outcomes than their White counterparts (2). Across low- and middle-income countries, CVD mortality disproportionately impacts those living in poverty, while Indigenous populations, Hispanic communities, and other historically marginalized groups face similarly elevated risks (5, 6). Importantly, these disparities are not the inevitable consequence of biological difference; they are the product of social, economic, and structural forces that shape the conditions in which people are born, grow, live, work, and age.

The social determinants of health (SDOH) – encompassing economic stability, neighborhood characteristics, education, community and social context, and healthcare system access – and the structural determinants that underpin them, including the policies, institutions, and systems that govern the distribution of power and resources, play a fundamental role in shaping cardiovascular risk profiles and disease progression (7, 8). Recent evidence demonstrates that social and structural determinants may be as predictive of cardiovascular disease as traditional clinical risk factors, with models incorporating only social, environmental, and behavioral drivers showing comparable or superior predictive accuracy to established CVD risk scores (9, 10). The World Health Organisation (WHO) defines social determinants of health as the non-medical factors that influence health outcomes, encompassing the broader set of forces and systems that shape the circumstances of daily life (11). Factors such as residential segregation, food insecurity, neighbourhood safety, and differential access to quality healthcare shape individual CVD risk through their influence on behaviors and biology (12). Moreover, systemic racism - the embedding of racial hierarchy across institutions, policies, and social norms - has been identified as a fundamental cause of cardiovascular health disparities (12). To address CVD disparities effectively, clinicians must be equipped to see and act upon these upstream determinants, not simply manage their downstream consequences.

Despite this mounting evidence, the integration of structural and social determinants into medical education curricula remains inconsistent and often episodic (13). Medical education plays a central role in this effort, yet most graduates feel underprepared to identify and address the social determinants affecting their patients (14). Historically, curricula have prioritised biomedical mechanisms and clinical management over the social and structural contexts of disease (15). Where social determinants have been taught, the dominant framework has been cultural competency - an approach focused on developing sensitivity to individual patients’ backgrounds and cultural practices (16). While valuable, structural competency is often a missing component of medical education and is a crucial framework that would enable students to recognise how systems and institutions shape the clinical encounter and produce differential health outcomes across populations (16).

Cardiovascular disease occupies a unique position as a teaching tool for structural competency in health professions education. Its prevalence and social distribution make it an ideal topic for discussions of health equity (17). Topics like hypertension, heart failure, myocardial infarction (MI), and stroke carry social histories that lend themselves to critical pedagogical exploration, allowing CVD to not only serve as a subject of clinical instruction but also as a lens through which structural determinants can be analysed (18). Health professions graduates are expected to address the determinants driving these disparities in their practice, yet the use of CVD as a teaching vehicle for structural and social competency remains poorly characterized in the educational literature. This gap represents a missed opportunity, as CVD’s unique position as a clinically and socially rich condition makes it a compelling vehicle through which structural competency teaching can be delivered.

While the epidemiological evidence linking social and structural determinants to cardiovascular outcomes is well-established, the evidence base for teaching this content through cardiovascular disease as a vehicle in health professions education remains nascent and poorly characterised. This systematic review aims to synthesise existing evidence on how health professions education uses cardiovascular disease as a teaching vehicle for structural and social determinants of health, and to evaluate the effectiveness of such interventions among health professions learners. Specifically, the review seeks to describe the pedagogical approaches, frameworks, and assessment strategies employed in CVD-based SDOH education and identify the barriers and facilitators to delivering this type of education across health professions curricula. By examining how educational institutions have operationalised this teaching, this review aims to help inform the development of more equitable, structurally informed cardiovascular curricula capable of preparing clinicians equipped to address these determinants through clinical practice, systems change, and advocacy.

## Methods

### Study design

This systematic review was registered with PROSPERO (registration number: CRD420251231266) and was conducted in accordance with the Preferred Reporting Items for Systematic Reviews and Meta-Analyses (PRISMA) guidelines (19). A PICOT framework was developed to guide the review, focusing on health professions learners across undergraduate, postgraduate, and continuing education settings. The primary outcomes of interest were the effectiveness of educational interventions incorporating cardiovascular disease as a teaching vehicle for structural and social competency, and the pedagogical approaches and frameworks used. Secondary outcomes included changes in learner self-efficacy and confidence in identifying and addressing health inequities, and the barriers and facilitators to delivering this education across health professions curricula. Given the anticipated heterogeneity in study designs, learner populations, intervention types, and outcome measures, a narrative synthesis approach was adopted to summarise and interpret findings across included studies, as quantitative pooling via meta-analysis was not considered feasible.

### Search strategy

A comprehensive electronic search was performed across the MEDLINE, Embase, CENTRAL, CINAHL, Pubmed, and Web of Science databases from January 1st, 2010, to March 13th, 2026. Search terms combined keywords and Medical Subject Headings related to health inequity and structural determinants of health, healthcare learners, and cardiovascular outcomes and diseases. Records obtained were uploaded to Covidence (Veritas Health Innovation, Melbourne, Australia) for screening, deduplication, and data management. The full search strategy is provided in Supplemental Table S1.

### Study selection

Studies were included if they were randomised controlled trials, non-randomised control studies, prospective or retrospective cohort studies, mixed-methods studies, qualitative studies, or cross-sectional studies that investigated educational interventions addressing the structural and social determinants of cardiovascular health among health professions learners. Studies were excluded if they were case reports, editorials, narrative reviews, or conference abstracts without accessible full texts. Only peer-reviewed articles published in English after January 1st, 2010, were included to ensure contemporary relevance to current medical education frameworks.

Screening of all records was conducted independently by two reviewers, who first reviewed titles and abstracts for relevance, followed by full-text assessments of all potentially eligible articles. Reasons for exclusion were documented. Disagreements regarding inclusion were resolved through discussion and consensus between the two reviewers.

### Data extraction

Data extraction was performed independently and in duplicate by two reviewers using a standardised data extraction form. Extracted information included study characteristics, learner demographics, and intervention details. Data relating to the role and integration of cardiovascular disease within the educational content were recorded, as well as the structural and social determinants of health focus of each intervention. Additionally, outcome data encompassing assessment methods, learner knowledge and attitudes were extracted.

### Methodological quality assessment

The methodological quality of all included studies was assessed using the Medical Education Research Study Quality Instrument (MERSQI) (20). The MERSQI evaluates study quality across six domains: study design, sampling, type of data, validity evidence for evaluation instrument scores, data analysis, and outcomes. Each domain is scored according to pre-specified criteria, with a maximum possible total score of 18. Published normative data indicate a median MERSQI score of 11.3 across medical education research studies (range 8.9–15.1) (20). Higher MERSQI scores reflect stronger study designs, more rigorous sampling, validated outcome measures, and more sophisticated data analysis, and are therefore indicative of higher methodological quality. Conversely, lower scores indicate methodological weaknesses—such as single-group designs without controls, reliance on non-validated self-report instruments, or absence of quantitative outcome data—that indicate lower methodological quality and limit the conclusions that can be drawn from those studies (20). As such, MERSQI scores were used to characterize the methodological quality of included studies, with scores below the published normative median of 11.3 indicating elevated methodological risk (20).

Each study was independently assessed by two reviewers using the MERSQI scoring criteria. Domain-specific scores were recorded for each study, and total scores were calculated by summing across all domains. Any discrepancies in scoring were resolved through discussion and consensus between the two reviewers. Full MERSQI domain scores and total scores for all included studies are presented in Table 1.

**Table 1:** Medical education research study quality instrument (MERSQI) scores for included studies (maximum possible score = 18; published normative median = 11.3).

MERSQI domain	Richardson et al. (21)	Moreira-Bouchard et al. (22)	Lewis and Tupas (23)	Berger and Harada (25)	Borgarelli et al. (24)	Maximum possible
Study design	1.5	1.5	1.5	1.0	1.0	3
Sampling: institutions	0.5	0.5	1.0	0.5	0.5	1.5
Sampling: response rate	1.5	1.0	1.5	1.5	0.5	1.5
Type of data	1.0	3.0	3.0	3.0	1.0	3
Validity evidence	0	1.0	1.0	1.0	0	3
Data analysis: sophistication	2.0	2.0	2.0	2.0	1.0	2
Data analysis: appropriate	1.0	1.0	1.0	1.0	1.0	1
Outcome	1.5	1.5	1.5	1.5	1.0	3
Total score	9.0	11.5	12.5	11.5	6.0	18

### Ethical statement

This systematic review utilised data exclusively from published studies and did not involve human participants. Therefore, it did not require ethical approval.

## Results

### Literature search

**Figure 1:**
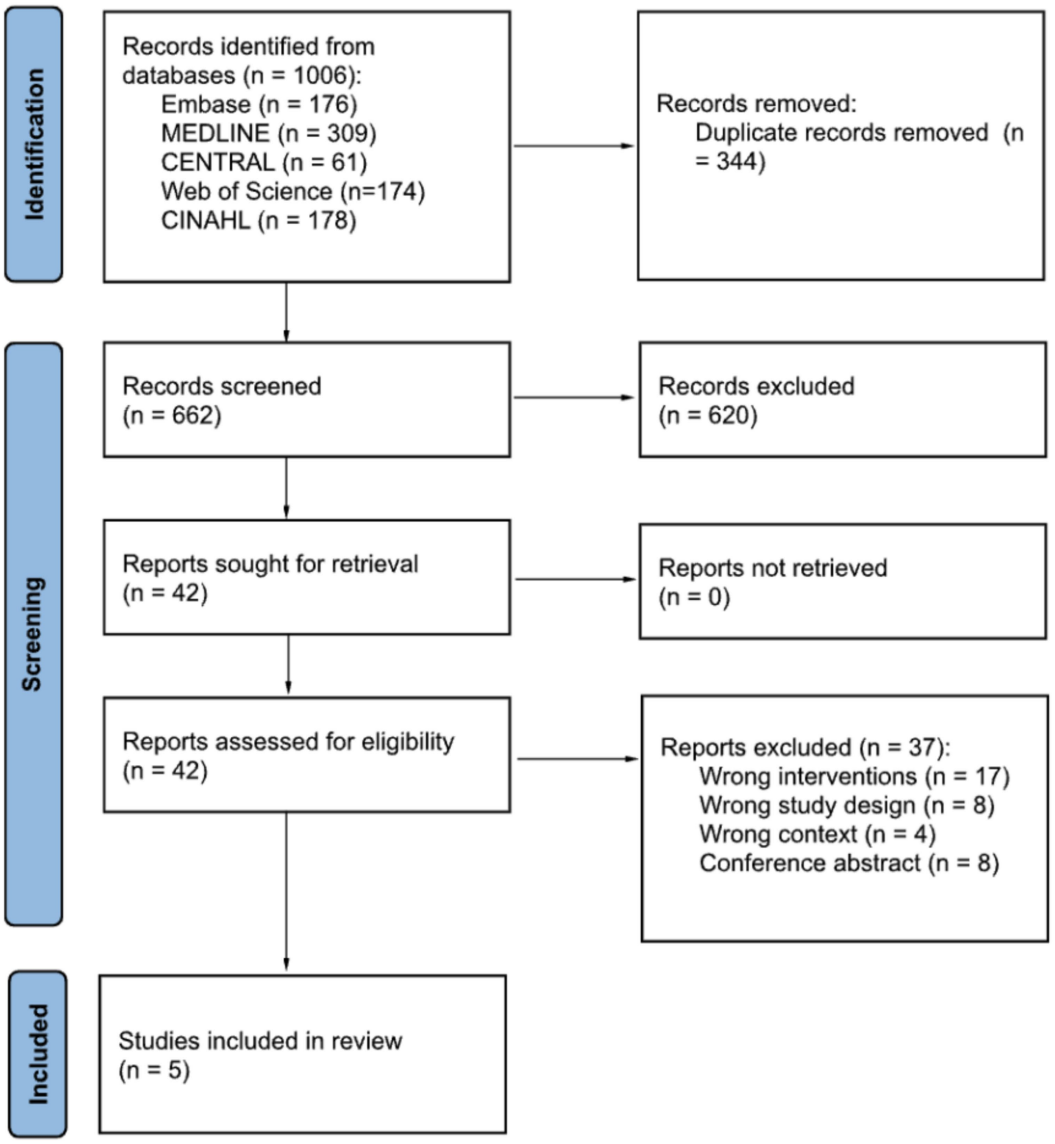
Preferred reporting items for systematic reviews and meta-analyses (PRISMA) flow diagram demonstrating the identification, screening, and inclusion phases of the systematic review.

A total of 1,006 publications were identified through comprehensive searches of six databases. After removing 344 duplicates, 662 records were screened; 620 were excluded based on title and abstract review. Of the 42 articles assessed for full-text eligibility, 37 were excluded (17 wrong interventions, 8 wrong study designs, 4 wrong context, and 8 conference abstracts), and 5 studies were included in the review (Figure 1).

### Study characteristics

Overall, 5 studies were included, with reported sample sizes ranging from 32 to 579 participants across four studies; one study did not report a sample size. All studies were conducted in the United States of America and spanned a range of health professions learners including medical students, pharmacy students, cardiovascular fellowship trainees, and pathophysiology students. Studies varied in design, comprising three pre-post educational interventions, one longitudinal curriculum evaluation study, and one curriculum development and implementation report. A summary of the characteristics of all included studies is presented in Tables 2, 3.

**Table 2:** Demographic characteristics of all studies included.

Study	Country	Study type	Sample size	Learner discipline
Richardson et al. (2024) (21)	USA	Pre-post interventional study	32 (14 M/18F)	Graduate Doctor of Medicine students
Moreira-Bouchard et al. (2025) (22)	USA	Pre-post interventional study	44 (12 M/30F/2NB)	Advanced undergraduate and early graduate students enrolled in an upper-level cardiovascular pathology course
Lewis and Tupas (2024) (23)	USA	Pre-post interventional study	93 (NR)	Graduate Doctor of Pharmacy students
Berger and Harada (2023) (25)	USA	Prospective curriculum evaluation	579 (NR)	Graduate Doctor of Medicine students
Borgarelli et al. (2026) (24)	USA	Educational innovation report	NR	Cardiovascular fellows

M, male; F, female; NB, non-binary; NR, not reported.

**Table 3:** Characteristics and key outcomes of included educational interventions.

Study	CVD topic	SDOH focus	Intervention type and duration	Key outcomes	Assessment method and outcome domain
Richardson et al. (2024) (21)	Hypertension	Race/racism, socioeconomic status, environmental exposures	Lunch-and-learn session (1 h)	100% of participants increased understanding of health inequities in hypertension; attitudes toward race-based guidelines shifted from neutrality toward disagreement	Pre-post survey assessing knowledge of health inequities in hypertension and attitudes toward race-based clinical guidelines
Moreira-Bouchard et al. (2025) (22)	Hypertension, myocardial infarction	Minority stress, structural racism, sex and gender minorities (SGM) and Black, Indigenous, and people of color (BIPOC) disparities	Single didactic lecture (75 min)	Significant improvements in mechanistic understanding of CVD disparities in SGM and BIPOC communities	Pre-post survey assessing mechanistic knowledge of CVD disparities and self-reported preparedness to address health inequities
Lewis and Tupas (2024) (23)	Hypertension	Structural racism, race-based vs. race-conscious medicine, socioeconomic status	Single didactic lecture + guided reading assignment (single session)	Significant improvements across all composite categories; high post-intervention open-ended knowledge score (mean 5.75/6)	Pre-post survey and open-ended knowledge questions assessing understanding, attitudes, beliefs, and confidence in applying SDOH concepts to clinical practice
Berger and Harada (2023) (25)	Myocardial infarction	Socioeconomic status, insurance status, neighbourhood, food access, race	Real patient learning curriculum (4 days)	Students demonstrated strong integration and application of structural concepts to real patient circumstances; curriculum consistently ranked among the highest course components	Rubric-assessed written reflections evaluating depth of structural reasoning and application of SDOH concepts to real patient circumstances
Borgarelli et al. (2026) (24)	Women’s CVD broadly	Sex/gender disparities, socioeconomic and psychosocial factors, intimate partner violence	Multimodal fellowship curriculum (full fellowship)	Positive trainee and faculty reception; no formal quantitative outcomes reported	Trainee and faculty feedback assessing perceived relevance and satisfaction with curriculum content

### Role of cardiovascular disease in the educational intervention

Cardiovascular disease served as the primary focus of the structural and social determinants content in four of the five included studies (21–24). In three of these, hypertension was the central topic through which structural and social determinants teaching was delivered (21–23). One study used racial disparities in hypertension prevalence and treatment as the clinical tool for exploring how social, economic, cultural, and environmental factors drive differential outcomes (21). Another similarly used hypertension pharmacotherapeutics as the vehicle for integrating structural determinants content, including a focus on structural racism and race-conscious compared to race-based medicine (23). A third used hypertension and MI as primary examples through which minority stress theory and its pathophysiological links to cardiovascular outcomes in sex and gender minorities (SGM) and Black, Indigenous, and people of colour (BIPOC) communities were taught (22). The curriculum aimed to educate fellows and addressed a broader range of women-specific cardiovascular conditions, including cardio-obstetrics, spontaneous coronary artery dissection (SCAD) and coronary microvascular disease (CMD), as the basis for an intervention that incorporated psychosocial, socioeconomic, and cultural determinants of women’s cardiovascular health (24). In contrast, one study used MI not as the primary focus but as a vehicle through which social determinants of health were taught to first-year medical students, with the explicit educational goal being SDOH learning (25). A summary of the CVD topics, SDOH focus, intervention types, and key outcomes for each study is presented in Table 3.

### Structural and social determinants content

Across all five studies, a range of structural and social determinants were incorporated into the educational content, though the extent to which varied considerably between studies. Race, racism, and ethnicity were the most consistently addressed determinants, appearing across four studies (21–23, 25). One intervention in particular examined the use of race as a biological category in clinical algorithms, challenging learners to consider how race-based treatment guidelines may exacerbate rather than reduce health inequity (21). Similarly, another study distinguished between race-based and race-conscious medicine, framing structural racism as a social determinant that operates independently of individual biological risk (23). A third study addressed racial health disparities in CVD through the lens of minority stress theory, linking structural stressors such as institutional racism and interpersonal discrimination to sympathetic nervous system activation and downstream cardiovascular risk (22).

Socioeconomic factors, including income, education, food access, neighbourhood environment, transportation, healthcare access, and health insurance status, were commonly addressed across multiple studies (21, 23, 25). One curriculum embedded these determinants within real patient circumstances, purposely including one insured and three uninsured MI patients to make visible to students the structural consequences of socioeconomic disadvantage and differential healthcare access (25). Uniquely, one study addressed the specific experiences of sexual and gender minority populations and BIPOC populations, examining how LGBTQIA2S+ identity and racial minoritisation acted as structural determinants of cardiovascular risk through minority stress, healthcare access inequities, and historical exclusion from clinical trials (22). The fellowship curriculum addressed factors disproportionately affecting women’s cardiovascular health, including intimate partner violence, socioeconomic challenges, and sociocultural roles, as components of a broader women’s cardiovascular health curriculum (24).

### Pedagogical approaches and intervention design

The included studies utilised a range of pedagogical approaches, spanning single-session didactic lectures, multiday experiential curricula, elective courses, and longitudinal multimodal fellowship programmes. Single didactic sessions integrated into existing courses were used by two studies (22, 23). One study delivered a single 75-min lecture integrated into a cardiovascular pathophysiology course, teaching minority stress theory as its framework (22). The other similarly integrated SDOH content into a single hypertension pharmacotherapeutics lecture, and was supplemented by a reading assignment and open-ended reflection questions (23).

A more intensive and multimodal approach was taken in one study, combining a two-week student elective, which involved a self-directed literature review on hypertension health disparities, with a one-hour “lunch-and-learn” session featuring professionally produced educational videos, interactive polls, and small group facilitated discussion (21). Another employed a four-day real patient learning curriculum in which first-year medical students interviewed real MI patients across two sessions, conducted assessments of the patients’ neighbourhoods and communities, and delivered formal presentations on the social determinants shaping their patients’ experiences to their classmates (25).

The most comprehensive of interventions included 15 mandatory one-hour lectures distributed throughout the fellowship training period, along with a one-month experiential component consisting of clinical rotations in specialist women’s cardiovascular health clinics, advanced cardiac imaging training, research engagement, and participation in community outreach events targeting underserved populations (24) (Table 3).

### Learner outcomes

Across the five included studies, learner outcomes were assessed using a range of methods, including self-reported questionnaires, open-ended knowledge questions, rubric-assessed written reflections, and informal qualitative feedback. Three studies employed pre- and post-intervention survey designs (21–23), one relied on post-intervention data in the form of written reflections and qualitative feedback (25), and one relied on trainee and faculty feedback (24). No study included longitudinal follow-up measures beyond the immediate post-intervention period.

All five reported improvements in at least one learner outcome following the educational intervention. Three of these demonstrated statistically significant improvements in learner knowledge or understanding (21–23). One study found that 100% of participants reported increased understanding of the impact of health inequities on hypertension, with statistically significant pre-to-post improvements across five knowledge items (21). Notably, attitudes toward race-based treatment guidelines shifted away from agreement and toward neutrality or mild disagreement post-session across all gender and racial groups, suggesting that the intervention prompted meaningful critical reflection on whether such guidelines truly advance health equity (21). Another study reported statistically significant improvements across all questionnaire categories, including understanding, perceptions, beliefs, and confidence, with the exception of one perception item, which was shown to be related to personal choice versus structural determinants (23). This study also showed that the post-intervention open-ended knowledge scores were high, with a mean of 5.75 out of 6, providing objective evidence that students could meaningfully apply SDOH concepts to cardiovascular clinical contexts following the intervention (23).

A third study found no change in students’ recognition that health disparities exist in SGM or BIPOC populations, as students already agreed with this at baseline (22). However, statistically significant improvements were observed in their understanding of the physiological mechanisms driving these disparities for both SGM (*p* < 0.001) and BIPOC (*p* < 0.001) communities, as well as in self-reported preparedness to design inclusive research studies (22). Assessment of student responses using Bloom’s taxonomy revealed that most students demonstrated only lower-order cognitive skills, with very few reaching the analysis level and none demonstrating synthesis, highlighting a key limitation of single-session delivery in achieving higher-order structural competency (22).

Another study with 579 students demonstrated that using real MI patients as a teaching vehicle produced consistently high-quality written reflections on structural determinants of health. The study showed that 90% and 96% of reflections met five or six of the six rubric criteria, assessing domains such as understanding of health disparities, socioeconomic influences on health, and goal setting across student cohorts in 2020–2021 and 2021–2022, respectively (25). A total of 97% of students agreed or strongly agreed that the curriculum was effective, and the intervention was consistently ranked among the top course components (25). Qualitative feedback highlighted the transformative impact of real patient narratives and students’ desire for greater community engagement (25).

The fellowship curriculum did not employ formal quantitative outcome assessment. Trainee and faculty feedback was positive, with fellows reporting that the curriculum was relevant to their future clinical practice, though suggestions included more frequent delivery (24) (Table 3).

### Barriers and limitations

Across all five studies, small sample sizes were a commonly reported limitation impacting statistical power and generalisability (21–23). The absence of long-term follow-up was noted in three studies, meaning that the sustainability of knowledge or attitude gains beyond the immediate post-intervention period could not be measured (21–23). Additionally, the reliance on self-reported outcomes and non-validated questionnaires was a limitation faced by some studies (21, 23), and an anonymous survey design in one study precluded paired individual-level analysis (22). One curriculum acknowledged that the patient pool lacked diversity, with all volunteer patients being male, potentially limiting the range of social determinants represented to students (25). Furthermore, the fellowship curriculum reported no formal quantitative assessment of knowledge or skills and relied on informal qualitative feedback, which limits the conclusions that can be drawn about educational effectiveness (24).

At the review level, several limitations warrant acknowledgment. First, the restriction to English-language publications may have excluded relevant studies conducted in other languages, introducing language bias. Second, the exclusion of conference abstracts and grey literature means that early-stage or unpublished work in this area may not be represented. Third, the high degree of heterogeneity across included studies—in learner type, intervention design, CVD topic, and outcome measures—precluded quantitative synthesis and limited the ability to draw pooled conclusions about intervention effectiveness.

### Methodological quality assessment

MERSQI total scores ranged from 6.0 (24) to 12.5 (23), with a median of 11.5. Three of the five studies (22, 23, 25) met or exceeded the published normative median of 11.3, indicating moderate to good methodological quality. Two studies scored below this threshold (21, 24), reflecting limitations in study design, absence of validated outcome measures, and reliance on informal qualitative feedback. Full domain-level scores are presented in Table 1.

## Discussion

### Integrating structural and social competency into cardiovascular health education

This systematic review provides promising evidence that cardiovascular disease represents a powerful and feasible teaching vehicle for embedding structural and social determinants of health (SDOH) competencies across diverse health professions learners. Five included studies collectively demonstrated that CVD-based SDOH education is not only achievable across various educational settings – from single-session interventions to comprehensive fellowship curricula – but that it consistently produces measurable improvements in learner knowledge, understanding, and structural reasoning (21–25). This finding is particularly significant given that the underlying epidemiologic evidence establishes an inextricable link between SDOH and cardiovascular outcomes. Models incorporating only social, environmental, and behavioral factors demonstrate predictive accuracy comparable to or exceeding traditional clinical CVD risk scores, underscoring that clinicians cannot adequately understand or address cardiovascular disease without grasping the upstream structural forces that shape its distribution (9).

The pedagogical approaches identified in this review align with established principles from the broader SDOH education literature, particularly the consensus that longitudinal, experiential, and community-engaged curricula produce deeper learning than didactic instruction alone (26–29). The most comprehensive intervention identified – a multimodal fellowship curriculum combining 15 mandatory lectures, clinical rotations in specialist women’s cardiovascular health clinics, advanced cardiac imaging training, research engagement, and community outreach – exemplifies this principle and reflects the longitudinal integration now recognized as foundational to effective SDOH education (24). Even more compelling, a single-session real patient learning curriculum with 579 medical students produced sophisticated levels of structural reasoning, with 90%–96% of written reflections demonstrating strong integration and application of structural concepts to real patient circumstances (25). These outcomes suggest that whether through intensive longitudinal approaches or strategically designed intensive experiences, CVD provides an inherently rich teaching platform whose clinical and social complexity lends itself naturally to exploration of health inequities.

### The unique role of cardiovascular disease as a structural competency teaching vehicle

Cardiovascular disease occupies a distinctive pedagogical position because its prevalence, social distribution, and heterogeneous presentations naturally invite critical examination of the forces that produce differential outcomes across populations (18). Race, racism, and ethnicity emerged as the most consistently addressed determinants across the included studies, with particular attention to how race-based clinical algorithms may inadvertently exacerbate rather than reduce health inequities (21–23, 25). Socioeconomic factors – encompassing income, education, food access, neighborhood environment, transportation, and healthcare access – were equally prominent, appearing across multiple studies and even appeared as embedded within real patient circumstances to make visible to learners the structural consequences of disadvantage (21, 23, 25). This learning tool specificity is important: rather than abstract discussions of health disparities, learners engaged with concrete cardiovascular case scenarios through which social determinants became tangible and clinically relevant (25). Drawing on the wider literature, broader conceptual frameworks—including structural competency, critical race theory, and health equity implementation frameworks—provide theoretical scaffolding that helps learners move beyond individual patient factors to interrogate institutional and societal determinants (30–32). This represents a fundamental shift from the historically predominant cultural competency paradigm, which focused on individual patient sensitivities, toward structural approaches that examine systems and institutions (32).

### Assessing learner outcomes and depth of learning

The evidence base reveals a consistent pattern: single-session interventions produce meaningful gains in foundational knowledge and awareness but have more limited impact on higher-order analytical competencies (21–23). One study applying Bloom’s taxonomy to assess learning depth found that most students demonstrated only lower-order cognitive skills following a 75-min lecture, with very few reaching analysis level and none demonstrating synthesis (22). However, this limitation should not be interpreted as a failure of single-session approaches; rather, it confirms that developing sophisticated structural competency requires sustained, progressive exposure (33). Three studies documenting significant improvements in knowledge, understanding, and confidence following single sessions demonstrate that even brief interventions provide value in building awareness – an essential foundation upon which deeper learning can be constructed (21–23). Notably, one study showed that attitudes toward race-based treatment guidelines shifted from neutrality toward slight disagreement post-intervention, suggesting—consistent with broader critical race theory frameworks—that educational encounters can prompt meaningful critical reflection on whether clinical tools truly advance health equity (21, 32). The diversity of measured outcomes – spanning knowledge, mechanistic understanding, confidence, self-efficacy, and demonstrated application to real circumstances – indicates that CVD-based SDOH education operates across multiple domains of learning, affecting not only what students know but how they think about structural determinants and how prepared they feel to engage with them in practice (21–25).

### Barriers, facilitators, and implementation imperatives

Despite these encouraging findings, significant barriers constrain widespread implementation of CVD-focused SDOH curricula (34–36). Small sample sizes and reliance on self-reported outcomes in the included studies limit statistical power and generalizability; notably, all five studies were conducted in the United States, limiting applicability to diverse international contexts. The absence of long-term follow-up data in most studies means that the sustainability of knowledge and attitude gains beyond the immediate post-intervention period remains unmeasured. Beyond methodological limitations, broader implementation challenges are well-documented in the literature: faculty members often lack training and comfort with teaching this content, curricula are perceived as space-limited, and SDOH concepts remain inadequately represented on certifying examinations (37–39). These challenges are not unique to CVD education but reflect systemic barriers in medical education more broadly (34, 36, 40). However, expert consensus demonstrates feasibility – a modified Delphi process achieved consensus that SDOH should comprise 29% of total curricula and be taught continuously throughout training (41). Successful implementation requires institutional commitment manifested through multiple pathways: dedicated personnel, clear evaluation metrics, faculty development grounded in critical pedagogy, protected teaching time, and meaningful community partnerships (35, 38, 39).

### Toward equitable, structurally informed cardiovascular curricula

Moving forward, medical schools seeking to advance SDOH integration into cardiovascular education should implement a systematic pathway grounded in institutional commitment, faculty development, curricular assessment, community partnership, longitudinal curriculum redesign, authentic assessment, and iterative improvement. Beyond the included studies, the broader medical education literature points to several critical components of effective CVD-SDOH curricula, including longitudinal integration across all years with progressive complexity, multiple teaching methods combining didactic content with skill-building and experiential learning, authentic community engagement, structural analysis that moves beyond individual factors to examine institutional and societal determinants, explicit anti-racism and health equity frameworks, interprofessional collaboration, and authentic assessment of knowledge, skills, attitudes, and demonstrated practice change (13, 41, 42). The current evidence base, while limited in scope and methodological rigor, provides sufficient foundation upon which institutions can build. Future research should prioritize curricula developed and evaluated in more countries, longitudinal outcome data measuring sustained behavior change and practice change, validated structural competency assessment tools, expansion to include broader CVD conditions, and implementation science frameworks to understand how to scale successful approaches across diverse institutional contexts. Ultimately, the integration of structural and social determinants through vehicles like cardiovascular health education is not merely an educational priority – it is a clinical and ethical imperative. Preparing health care providers who understand CVD not simply as a collection of individual risk factors but as a condition fundamentally shaped by upstream structural forces, and who are equipped to address these determinants through clinical practice, systems change, and advocacy, is essential for developing a health workforce capable of achieving health equity.

## Conclusion

This systematic review suggests that cardiovascular disease is a feasible and effective teaching vehicle for structural and social competency across diverse health professions learners. The studies included consistently showed meaningful improvements in SDOH knowledge, mechanistic understanding, and structural reasoning following CVD-based educational interventions, though findings are limited by small study numbers, US-only settings, and short-term outcome measures. The range of learner populations and pedagogical formats represented across the included studies reflects the versatility of CVD as a platform for SDOH education, and establishes a foundation upon which future research and curriculum development can build toward a more structurally competent health workforce.

## Data Availability

The original contributions presented in the study are included in the article/Supplementary material, further inquiries can be directed to the corresponding author.
