# Supplementary material for: Embedding structural and social determinants of cardiovascular health into medical education: a systematic review of pedagogical frameworks
Source: Front Med (Lausanne). 2026 Jul 13;13:1860983. doi: 10.3389/fmed.2026.1860983 (PMC13402413; doi:10.3389/fmed.2026.1860983)
Supplement: Supplementary file 1 [file Table_1.docx]

**Supplemental Table S1.** Full search strategy used across databases.

Detailed search strategy used to identify eligible studies examining the use of cardiovascular disease as a teaching vehicle for the structural and social determinants of health in health professions education. The strategy was adapted for each database (MEDLINE, Embase, CENTRAL, CINAHL, PubMed, and Web of Science) using both keywords and Medical Subject Headings terms. Searches were performed on March 13, 2026.

| Entry | Central | MEDLINE | EMBASE | Pubmed | CINAHL | Web of Science |
| --- | --- | --- | --- | --- | --- | --- |
| (  "social determinants":ti,ab  OR "structural determinants":ti,ab  OR "health inequity":ti,ab  OR "health disparit*":ti,ab  OR "structural competency":ti,ab  OR "social justice":ti,ab  ) | 5,276 | 78,469 | 65,398 | 65,415 | 41,816 | 119,689 |
| (  "medical student*":ti,ab  OR "healthcare student*":ti,ab  OR "health professions student*":ti,ab  OR "medical education":ti,ab  OR "healthcare education":ti,ab  OR "health professions education":ti,ab  OR "health education":ti,ab  OR pedagogy:ti,ab  OR pedagogical*:ti,ab  OR "case-based learning":ti,ab  OR "experiential learning":ti,ab  OR "advocacy training":ti,ab  ) | 34,834 | 252,638 | 227,142 | 197,375 | 110,060 | 408,452 |
| (  cardiovascular*:ti,ab  OR cardiac*:ti,ab  OR cardiolog*:ti,ab  OR CVD:ti,ab  OR "heart disease":ti,ab  OR hypertension:ti,ab  OR "myocardial infarction":ti,ab  OR "heart failure":ti,ab  OR stroke:ti,ab  ) | 323,516 | 1,983,174 | 3,567,681 | 2,370,549 | 601,773 | 3,145,199 |
| 1 AND 2 AND 3 | 61 | 309 | 176 | 108 | 178 | 174 |
